# Rapid Population Growth in Chinese Floodplains from 1990 to 2015

**DOI:** 10.3390/ijerph15081602

**Published:** 2018-07-28

**Authors:** Yongqiang Fang, Shiqiang Du, Paolo Scussolini, Jiahong Wen, Chunyang He, Qingxu Huang, Jun Gao

**Affiliations:** 1 Department of Geography, Shanghai Normal University, 100 Guilin Road, Shanghai 200234, China; ffyq1994@126.com (Y.F.); jhwen@shnu.edu.cn (J.W.); gaojun@shnu.edu.cn (J.G.); 2 Institute for Environmental Studies, Vrije Universiteit Amsterdam, 1081 HV Amsterdam, The Netherlands; paolo.scussolini@vu.nl; 3 State Key Laboratory of Earth Surface Processes & Resource Ecology, Beijing Normal University, Beijing 100875, China; hcy@bnu.edu.cn (C.H.); qxhuang@bnu.edu.cn (Q.H.); 4 Institute of Urban Studies, Shanghai Normal University, 100 Guilin Road, Shanghai 200234, China

**Keywords:** floodplain, population exposure, flood memory, sustainability, China

## Abstract

Although China suffers from frequent and disastrous floods, the spatiotemporal pattern of its population living in the floodplain (PopF) is still unknown. This strongly limits our understanding of flood risk and the effectiveness of mitigation efforts. Here we present the first quantification of Chinese PopF and its dynamics, based on newly-available population datasets for years 1990, 2000, 2010, and 2015 and on a flood map. We found that the PopF in 2015 was 453.3 million and accounted for 33.0% of the total population, with a population density 3.6 times higher than outside floodplains. From 1990 to 2015, the PopF increased by 1.3% annually, overwhelmingly faster than elsewhere (0.5%). A rising proportion (from 53.2% in 1990 to 55.6% in 2015) of the PopF resided in flood zones deeper than 2 m. Moreover, the PopF is expected to increase rapidly in the coming decades. We also found the effect of flood memory on controlling PopF growth and its decay over time. These findings imply an exacerbating flood risk in China, which is concerning in the light of climate change and rapid socioeconomic development.

## 1. Introduction

Flood exposure refers to people, infrastructure, housing, production capacities, and other human assets that are located in floodplains [1,2]. It is therefore a result of the combination of physical environment (i.e., the geographical extent of the hazard) and socioeconomic factors (e.g., distribution of population and assets) [3,4,5]. Population in floodplain (PopF) is an important component of flood exposure [6,7]; its variation is increasingly considered as a major driver of disastrous flood consequences [1,8,9,10]. The global PopF of a 100-year return period flood, i.e., which is expected to occur once per century, increased approximately twofold from 1970 to 2010 [10]. Even in countries with a long tradition of flood management, like the Netherlands, PopF increased dramatically in the last decades [11]. Monitoring changes in PopF is thus essential for understanding the dynamics of flood risk at all scales [12,13,14].

China is one of the countries that most suffer from disastrous floods [1,15,16,17]. Between 1990 and 2015, a total of 162 riverine floods occurred in China, aggregately killing 23,990 people and affecting 1.7 billion, with a total economic loss of 191 billion USD [18]. On the other hand, China has been experiencing unprecedented socioeconomic development since the late 1970s, with an average annual GDP growth of 9.7% between 1990 and 2015 [19]. It is reasonable to hypothesize that this has accelerated floodplain development and led to PopF growth [20,21]. Assessing PopF dynamics becomes more important for managing flood risk and promoting society sustainability because the PopF amount primarily determines a region’s flood protection level in China [22].

Most previous studies have provided regional estimations of the PopF in China. For example, Zhang et al. [23] found that population grew rapidly by 129,900 or 33.6% during 1990–2005 in the Jingjiang flood diversion area of the middle Yangtze River basin. Based on a historical flood database, Huang et al. [24] found that more than 40% of the total 2010 population of Jiangsu, Zhejiang, and Shanghai would be exposed to the most extreme floods of the period 1644–1949. Wang et al. [25] revealed a significant increasing trend in the exposed population during 1984–2011 in the Jiangsu Province. Liang et al. [12] found that, during 1990–2010, population increased in Chinese counties that showed a significant increasing trend in rainstorm frequency.

However, a national-scale study of PopF is still to be accomplished in China for two major reasons. First, a national scale floodplain map was not available [26]. In previous studies, floodplains were approximated by flood diversion areas [23], historically affected counties [24,25], or areas with a certain rainstorm frequency [12]. However, flood diversion areas are only a small portion of floodplains in China. Regarding historically flooded counties, their PopFs are generally different from the total population because a county is typically not homogeneously prone to floods. Moreover, population in rainstorm-prone counties is hardly a reliable indicator of PopF because rainstorm is neither a sufficient nor a necessary condition to induce floods, especially in the context of the very large river systems in China, where floods may manifest downstream and vary from sites of most intense rainstorm. Second, a reliable space-explicit dataset of population in China was previously scarce [27]. Previous studies mainly employed county-level census data for analyzing PopF but ignored the unequal distribution of population within counties.

A national-scale PopF analysis is now feasible because of two recently released datasets. First, a global flood map produced by the CIMA foundation [28] has the adequate resolution to represent floodplains in China. Second, the China Temporal Datasets of Harvard Dataverse was released by the WorldPop program [29]. This dataset is suitable for analyzing population change across time and has been applied, e.g., to analyze farmland transition and grain production [30]. This study assesses the patterns and dynamics of PopF in China during 1990–2015 by using these two datasets.

## 2. Materials and Methods

### 2.1. Data Source

**Figure 1:**
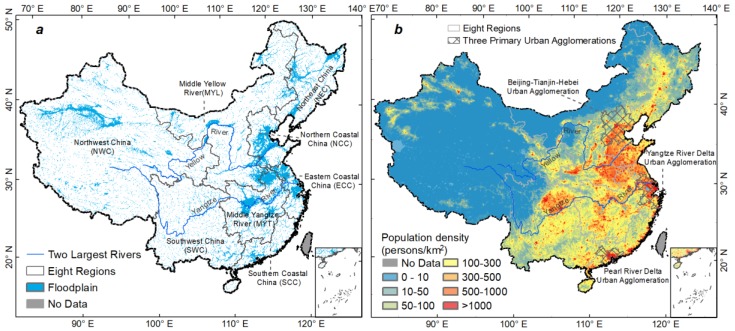
100-year river floodplain (**a**) and population distribution in 2015 (**b**).

To characterize the floodplain, we employed a riverine flood depth map with a 100-year return period and a spatial resolution of 1 km. It was provided by the CIMA foundation and is publicly accessible from the Global Risk Data Platform (http://preview.grid.unep.ch/). This dataset was produced based on regional river flow frequency analysis and hydrodynamic models, and validated against historical floods [28]. It has been used for analyzing global flood risk [1] and studying floodplain urbanization in China [21]. Flood areas are mainly distributed in the middle and lower reaches of large rivers in eastern part (Figure 1a), which are also the most populous areas in China (Figure 1b).

To represent the exposed population, we adopted the China Temporal Dataset of Harvard Dataverse, which was produced by the WorldPop program [29] and is accessible at http://www.worldpop.org.uk. This population density dataset was disaggregated from county-level population census data to a grid level by a random forest regression and dasymetric mapping technique. It was validated against census data at township scale. This dataset temporally covers 1990, 2000, 2010, and 2015. It originally had a spatial resolution of 100 m and was aggregated to 1 km resolution to match the flood map. Because this dataset did not include Tibet, we filled this gap with the gridded population of the world, version 4 (GPWv4) [31].

To evaluate the quality of the CTDHD data, a township level census data of 2010 was employed, which was released from the Population Census Office of China [32]. It is the most detailed dataset that is publicly accessible. At the township level, the CTDHD and the Population Census Office data are strongly correlated (R^2^ = 0.88, *p* < 0.001), which is consistent with the data producer’s accuracy report (R^2^ = 0.85, 0.88, and 0.86 for 1990, 2000, and 2010, respectively). In contrast, the GPWv4 [31] and the Landscan [33], two popular global scale gridded population data, have much weaker correlation with the Population Census Office data, R^2^ = 0.81 and R^2^ = 0.72, respectively. Therefore, the CTDHD population dataset is more reliable for China than the widely used global data GPWv4 and Landscan.

### 2.2. Calculate PopF and Its Indices

Floodplain was defined as the maximum extent (or flood depth > 0 m) of the 100-year flood (Figure 1a), which is consistent with the flood risk assessment by Shi and Roger [34] and the flood exposure analysis by Jongman, et al. [10] and Du, et al. [21]. PopF was then calculated by overlaying the floodplain and the population datasets using ArcGIS 10.3 (Esri, Redlands, CA, USA).

To describe the patterns of PopF, we used Equation (1) to calculate the PopF density, which is the ratio of PopF to the area of floodplain (FP) in a spatial unit i:

(1) $$\mathrm{PopF}\;\mathrm{density}(i)=\frac{\mathrm{PopF}}{\mathrm{FP}}$$

Similarly, we also calculated the population density outside the floodplain. In addition, the annual growth rate was calculated for PopF and population outside floodplain during 1990–2015 following Equation (2):

(2) $$\mathrm{annual}\;\mathrm{growth}\;\mathrm{rate}(\%)=(\sqrt[{\left(t_{2}-t_{1}\right)}]{\frac{x_{t2}}{x_{t1}}}-1)\times 100$$

where x_t1_ and x_t2_ refer to the PopF or to the population outside the floodplain in years t_1_ and t_2_, respectively.

### 2.3. Analyzing PopF at Multiple Scales

We analyzed the patterns and changes of PopF at three scales, i.e., country, region, and urban agglomeration. To recognize regional differences in geography and socioeconomic conditions, a delineation of eight regions was employed according to the Coordinated Regional Development Strategy and Policy Reports of China [35]: Northern Coastal China (NCC), Eastern Coastal China (ECC), Southern Coastal China (SCC), Middle Yangtze River (MYT), Middle Yellow River (MYL), Northeast China (NEC), Northwest China (NWC), and Southwest China (SWC) [36] (Figure 1a). Additionally, we analyzed the PopF and its changes in the three primary urban agglomerations in China [37]: Beijing-Tianjin-Hebei, the Yangtze River Delta, and the Pearl River Delta (Figure 1b).

## 3. Results

### 3.1. Population in the Floodplain in 2015

The 100-year floodplain covers 12.1% (or 1.1 million km^2^) of land area in China, exposing 33.0% of the population of 2015, or 453.3 million people. The density of PopF is on average 396 people/km^2^, 3.6 times of that outside floodplains (111 people/km^2^).

**Figure 2:**
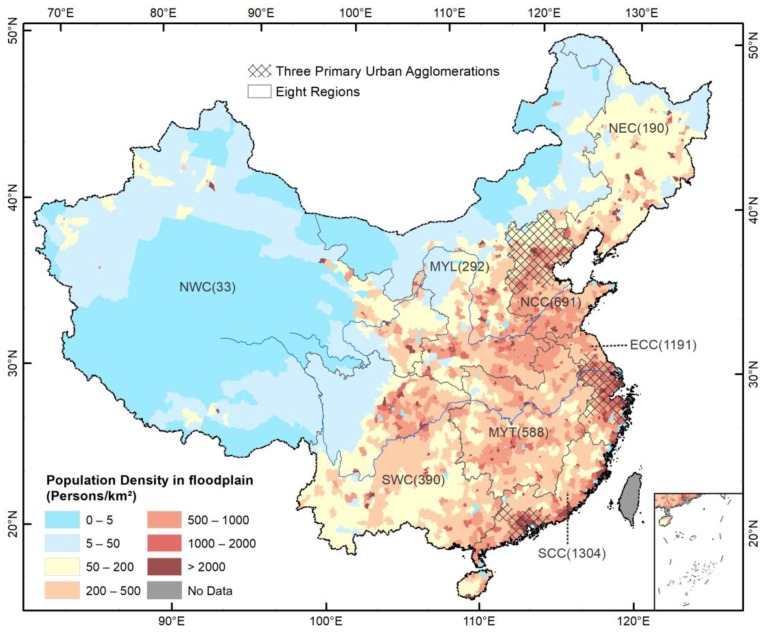
Density of population in the floodplain in China in 2015. Note: numbers in bracket are the average density for regions (in people/km^2^); refer to Figure 1 for the abbreviations of the regions.

The PopF shows high spatial heterogeneity (Table 1, Figure 2). A majority (72.6%, or 328.9 million) of the national PopF is located in NCC, ECC, SCC, and MYT, which only account for 35.5% of China’s floodplain. The average PopF density of these four regions is 810 people/km^2^, two times of the national average; in contrast, the PopF density for the other four regions is only 169 people/km^2^, or 42.6% of the national average (396 people/km^2^). The region with the highest PopF density (1304 people/km^2^) is SCC, accounting for 12.6% of China’s PopF but for only 3.8% of the floodplain area.

The PopF is further concentrated in the three primary urban agglomerations (Figure 2). The urban agglomerations jointly host a PopF of 136.5 million, accounting for 30.1% of the PopF in China; in contrast, they only have 9.2% of China’s total floodplains. The PopF density is as high as 1292 people/km^2^, or 3.3 times of the national average.

### 3.2. Changes in PopF between 1990 and 2015

**Figure 3:**
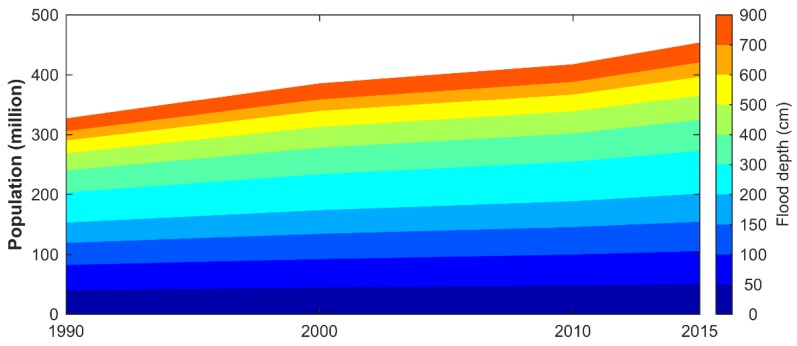
Population in Chinese floodplain for different flood depths.

The total PopF in China increased by 38.8% from 326.5 million in 1990 to 453.3 million in 2015. The annual PopF growth was 1.3%, while the population outside floodplain only increased by 0.5% annually (Table 1). As a result, the ratio of PopF to China’s total population increased from 28.6% in 1990 to 33.0% in 2015. Moreover, PopF boomed uniformly across different flood depths (Figure 3). In the zones with flood depths larger than 2 m, where damage ratio is high and can range from 41–100% [11], the PopF grew even faster and its ratio to the total PopF increased from 53.2% in 1990 to 55.6% in 2015.

**Figure 4:**
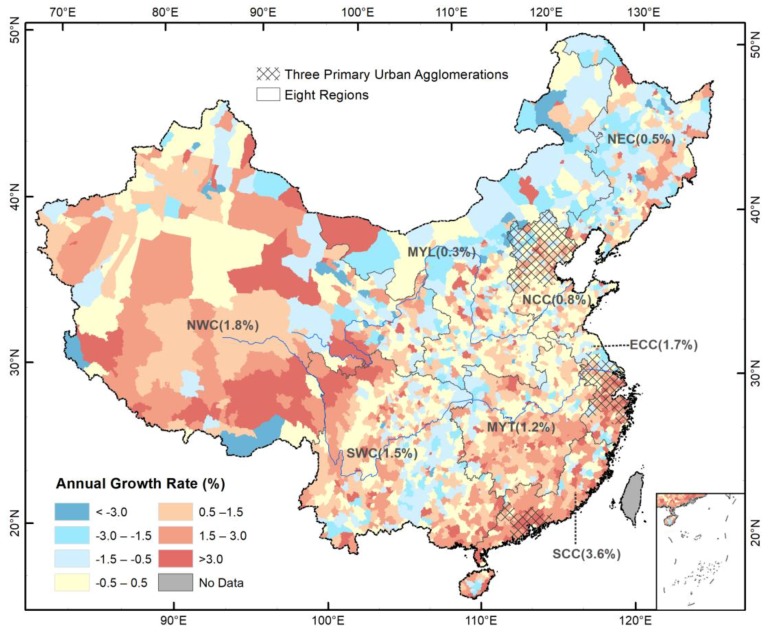
The annual growth rate of population in the floodplain during 1990–2015. Note: refer to Figure 1 for the abbreviations of the regions.

A majority (82.4% or 104.5 million) of the PopF growth occurred in NCC, ECC, SCC, and MYT (Figure 4). The annual growth rate of the PopF in the four regions was 1.5%, higher than the national average of 1.3%. Particularly, SCC and ECC experienced a PopF growth of 33.4 million and 32.5 million and an annual growth rate of 3.6% and 1.7%, respectively. NWC and SWC also experienced high annual growth rates of 1.8% and 1.5%, respectively; in contrast, the PopF only increased by 0.3% and 0.5% annually in MYL and NEC, respectively. A large portion of the PopF growth (59.0 million people, 46.5% of the total PopF increase in China) was further clustered in the three primary urban agglomerations (Figure 4), where the annual growth rate was 2.3%, significantly higher than the national average (1.3%).

### 3.3. Heterogeneous Trends of PopF during the Last Decades

**Figure 5:**
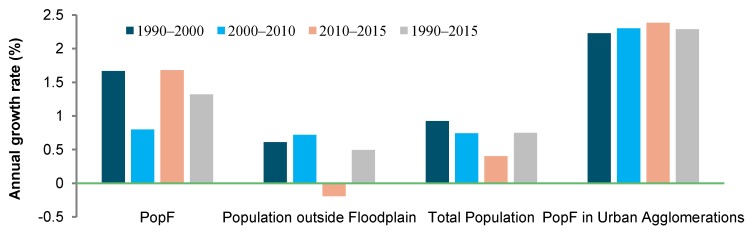
The annual growth rate of population in the floodplain (PopF), population outside floodplain, total population, and PopF in the three primary urban agglomerations.

Demographic changes in China’s floodplain were not uniform during the study period (Figure 5). While the annual growth rate of total population decreased from 0.9% during the 1990s to 0.4% during the 2010s (2010–2015), the PopF followed a different evolution: it grew faster in the 1990s and 2010s (both 1.7% annually) than in the 2000s (0.8% annually). By contrast, the population growth rate outside the floodplain slightly increased from 0.6% in the 1990s to 0.7% in 2000s and then dropped to −0.2% in the 2010s.

At the regional scale, PopF growth slowed down from 1990s to 2000s in six of the eight regions, but accelerated in NCC and ECC. Most prominently, the annual growth rate of PopF decreased in SWC from 2.4% during 1990s to −0.2% during 2000s and in MYT from 1.6% to 0.1%. However, the 2010s witnessed an accelerated PopF growth in five of the eight regions, except NEC, NCC, and MYL. By contrast, the PopF in the three primary urban agglomerations of BTH, YRD, and PRD maintained a high growth rate across all the three periods, even consistently accelerating from 2.2% in 1990s to 2.4% in 2010s.

## 4. Discussion

### 4.1. PopF Growth and Its Connection to Flood Risk

The fast PopF growth aggravates flood risk in three ways. First, PopF growth directly increases flood exposure [11]. In our study, PopF boomed across different flood depths uniformly. With a rising proportion (from 53.2% in 1990 to 55.6% in 2015) of the PopF residing in the zones exposed to floods deeper than 2 m, huge potential flood damage and exacerbating flood risk are estimated for China. Second, the rising share of PopF to total population (from 28.6% in 1990 to 33.0% in 2015) can compromise the capacity to cope with flood damage and exacerbate the post-disaster need of relief resources, intervention, and recovery assistance [11]. Particularly, PopF growth will aggravate flood risk in northwest China because its flood defense systems are relatively poor [20,38]. Third, the PopF growth can also aggravate flood risk because floodplain development alters ecological systems and hampers their flood regulation function [39]. For example, the rapid growth of PopF in middle Yangtze River reaches caused lakes to shrink, with negative consequences for flood control [39].

According to the ‘World Population Prospects: the 2017 Revision’ [40], the total population in China can reach 1.5 billion in 2030. If the proportion of PopF to total population in 2030 is similar to that in 2015, the PopF will increase by approximately 45.3 million, or by 10.0%, from 2015 to 2030. If the proportion of PopF to total population continues to rise linearly, then the PopF will grow by 74.3 million, or 16.4% during 2015–2030. Therefore, there will potentially be an exacerbating flood risk in China if the rapid PopF growth is not properly controlled.

### 4.2. Policy Implications

A review of relevant policies (Table 2) reveals that China has nearly considered all possible measures to control floods. Specifically, authorities at multiple scales built dams and levees, established flood diversion zones, relocated people, restored and protected wetlands, and required integration of flood control into urban planning [21,41,42,43,44]. However, the fast PopF growth hints that not all the measures have been effectively implemented. A survey in 2013 [38] even found that 50% (or 321) of the 642 Chinese cities did not reach the required flood control standards; moreover, 44% (or 284) of the cities did not complete or update flood control plans, increasing from a number of 170 cities in 2006. Neglecting to upgrade flood protection reduces the cost of floodplain development, therefore increasing PopF and aggravating flood risk. Given that the flood protection level of a Chinese region is primarily determined by its PopF amount [22], flood protection systems should be adjusted to reflect PopF dynamics.

Moreover, all the PopF control efforts were further hampered by a lack of flood hazard maps at a national scale [26]. Without flood hazard maps, policy makers may not be aware of flood risk. This can even lead to policies increasing PopF growth as a side effect. For example, the “population relocation project for poverty alleviation” policy (2008), which mainly aimed to relocate residents of poverty from mountainous areas to flat areas to alleviate their poverty, may partially explain the PopF increase in northwest and southwest China from 2010 to 2015. In 2011, the “China’s National Plan of Integrated Disaster Prevention and Reduction (2011–2015)” required to produce flood hazard maps at different scales, which will play a vital role for an effective control of PopF growth in the future. We further recommend making the maps publicly accessible to enable public participation and raise risk awareness.

The slowdown of PopF growth in the 2000s suggests that flood memory had played a role. Memory of disastrous floods can dramatically increase risk awareness and influence decision making [45]. After the disastrous flood of 1998, several policies were released to mitigate flood hazard [41] (Table 2). Policies of 1999 included “convert farmland back to lake”, “removing polder dykes for floodwaters”, and “relocating people of polders to new towns” [46,47,48] (Table 2). In Yangtze River Basin alone, these policies have relocated at least 2.4 million PopFs to adjacent villages on higher land [48]. From our results, these policies seem to have achieved an effect during the 2000s, as PopF growth decreased in six of the eight regions. However, this flood memory effect seems to have dissipated over time, as China’s PopF growth rate accelerated again after 2010 at both national and regional scales. Therefore, risk education and awareness should be enhanced to counteract the declining flood memory, which also links with the accessibility of flood hazard maps.

### 4.3. Uncertainty and Future Perspectives

Population distribution maps with high resolution are critical for flood exposure analysis. Dasymetric mapping has been widely applied to disaggregate population data from census unit into a grid level [6,49]. However, uncertainties are inevitable in the disaggregated population data. Although it fits census data better than Landscan and GPWv4, the China Temporal Datasets of Harvard Dataverse (CTDHD) is no exception. We hope population distribution data can be further improved with the development of open source data and volunteered geographic information, e.g., the OpenStreetMap [50]. Uncertainty is also associated with the flood map. We expect that the flood map can be further improved by incorporating flood defense data into analyses and by reinforcing flood models [28,51]. In line with recent development of large-scale flood modeling, output from multiple models should be analyzed in the future to provide an estimation of uncertainties that originate from different model setups and assumptions [52]. To control uncertainties, we presented results at the macro scales (i.e., country, the eight regions, and urban agglomerations) rather than at a grid level. Therefore, our main findings should be robust towards the uncertainties of the employed data. Additionally, the PopF analysis should be extended by looking at the future, when variations are expected in population distribution, due to socioeconomic development [1,10,16], and in flood hazard, due to climate change [51].

## 5. Conclusions

The paper revealed a concentration of the population in floodplains (PopF) in China and its rapid growth between 1990 and 2015, according to the most updated population dataset and flood map. First, PopF accounted for 33.0% (or 453.3 million) of China’s total population with a population density 3.6 times of that outside floodplains. Second, PopF grew substantially faster (annually 1.3%) than elsewhere and accelerated recently. Third, a rising proportion (from 53.2% in 1990 to 55.6% in 2015) of the PopF was exposed to floods deeper than 2 m. Fourth, the PopF is expected to increase by another 10.0–16.4% from 2015 to 2030. The rapid PopF growth suggests an exacerbating flood risk in China and the need to adjust the flood protection system accordingly. Flood maps at different scales should be developed and made publicly accessible to enable devising local-scale risk reduction and PopF control strategies. Moreover, we detected the effect of flood memory on policy, but also its dissipation in time. This phenomenon could be addressed by raising awareness of citizens and stakeholders to flood risk.

## Figures and Tables

**Table 1:** Population in the floodplain (PopF) and its density in 2015 and growth during 1990–2015 in different regions of China.

Region	PopF (million)	PopF Density (people/km^2^)	PopF Growth (million)	PopF Annual Growth Rate (%)
Northern Coastal China (NCC)	76.6	691	13.2	0.8
Eastern Coastal China (ECC)	93.3	1191	32.5	1.7
Southern Coastal China (SCC)	57.1	1304	33.4	3.6
Middle Yangtze River (MYT)	101.9	588	25.4	1.2
Middle Yellow River (MYL)	53.0	292	4.1	0.3
Northeast China (NEC)	24.0	190	2.8	0.5
Northwest China (NWC)	11.1	33	4.0	1.8
Southwest China (SWC)	36.3	390	11.5	1.5
China	453.3	396	126.8	1.3

**Table 2:** Major policies related to the population in floodplain (PopF) in China during 1989–2011. (Expanded from [21]).

Year	Policy/Decree	Main Contents	Scale	Administrations
1989	Urban Planning Law of China	Flood protection measures should be implemented for areas that are prone to catastrophic floods.	National	MHURD
1998	Opinions on restoring rivers and lakes and reinforcing flood defense system after Great Flood of 1998	“Convert farmland back to lake”, “removing polder dykes for floodwaters”, and “relocating people of polders to new towns” should be implemented to restore the drainage capacity of the river-lake system and to reduce flood risk.	Regional	MWR, MCA, MHURD
1998	China’s National Disaster Reduction Plan (1998–2010)	Hydraulic projects should be constructed for comprehensive disaster reduction; core cities should construct flood protections.	National	MHURD, MCA, MWR
1999	Report on “convert farmland back to lake” and “removing polder dykes for floodwaters” in Yangtze River basin	Accomplishing “convert farmland back to lake”, “removing polder dykes for floodwaters”, and “relocating people of polders to new towns” in 3–5 years in Hubei, Hunan, Jiangxi, and Anhui provinces.	Regional	MWR, MCA
2000	Compensating measures for the acquisition of flood diversion areas	Control the population growth in flood diversion areas and organize planned emigration	Regional	MWR, MCA
2007	China’s National Plan of Integrated Disaster Reduction (2006–2010)	Disaster reduction should be considered in land use, urban, and post-disaster reconstruction plans; a national integrated disaster risk map should be implemented.	National	MHURD, MCA, MWR
2008	Population relocation project for poverty alleviation	Relocate impoverished residents from mountainous areas to flat areas and provide them fertile lands and work opportunities to alleviate poverty	Regional	NDRC
2011	China’s National Plan of Integrated Disaster Prevention and Reduction (2011–2015)	Hydraulic projects should be constructed and reinforced for flood prevention, particularly for middle- and small-sized rivers; disaster prevention and reduction should be integrated with regional development plans; integrated risk maps of different scales should be produced.	National	MHURD, MWR, MCA

Abbreviations: MHURD: Ministry of Housing and Urban-Rural Development; MWR: Ministry of Water Resources; MCA: Ministry of Civil Affairs; NDRC: National Development and Reform Commission.
